# Perioperative complications and mid-term outcomes in total hip and knee joint arthroplasty among solid organ transplant recipients: lowest reoperation-free survival and patient survivorship in lung transplant recipients

**DOI:** 10.1007/s00402-025-05836-6

**Published:** 2025-04-11

**Authors:** Dominic Simon, Jennifer Kalil, Maximilian Lerchenberger, Lennart M. Schroeder, Horst Balling, Wolfgang Böcker, Boris M. Holzapfel, Jörg Arnholdt, Gautier Beckers

**Affiliations:** 1 Department of Orthopaedics and Trauma Surgery, Musculoskeletal University Center Munich (MUM), University Hospital, LMU Munich, Marchioninistr. 15, 81377 Munich, Germany; 2 Department of Surgery, Royal Victoria Hospital, Mcgill University Health Center, 1001 Blvd Decarie, Montreal, QC H4A 3J1 Canada; 3 Cancer Research Program, Mcgill University Health Center, Research Institute, 1001 Blvd Decarie, Montreal, QC H4A 3J1 Canada; 4 Center for Spine Surgery, Neckar-Odenwald-Kliniken gGmbH Buchen, Dr.-Konrad-Adenauer-Str. 37, 74722 Buchen, Germany

**Keywords:** Total hip arthroplasty, Total knee arthroplasty, Solid organ transplantation, Complications, Implant survivorship

## Abstract

**Introduction:**

Performing total joint replacements (TJR) in patients with solid organ transplantations (SOT) is associated with an increased risk of complications and reoperation. The aim of this study is to report on implant survivorship, patient survivorship, and complication rates for total knee arthroplasty (TKA) and total hip arthroplasty (THA) performed in heart, lung, liver and kidney transplant recipients.

**Materials and methods:**

Forty patients with heart, lung, liver, or kidney transplants who underwent primary THA or TKA between January 1, 2013, and July 31, 2023, were included. Implant survivorship, reoperation-free survivorship, patient survivorship, and complication rates were compared between the subgroups.

**Results:**

At a mean follow-up of 5.18 years, Implant survivorship and reoperation-free survival for the entire cohort at the last follow-up were 97.5% and 85%, respectively. Kaplan–Meier survival estimates demonstrated 5- and 10-year reoperation-free survival rates of 86.5% (95% CI: 76%–98.4%) and 57.6% (95% CI: 25.6%–100%), respectively. The lung transplant group had the shortest reoperation-free survival, although not statistically significant (p = 0.07), a significantly higher risk of reoperation, with a hazard ratio (HR) of 6.9 (95% CI: 1.1–41.2, p = 0.04) and both the lowest 5-year patient survivorship at 68.6% (p = 0.04) and the highest risk of death after TJR with a HR of 7 (95% CI: 1.2–45.5, p = 0.03).

**Conclusion:**

Patients with SOT exhibit excellent mid-term implant survivorship, with a rate of 97.5%. Lung transplant recipients show the lowest rates of both reoperation-free survival and overall patient survivorship compared to heart, kidney, and liver transplant recipients. Despite this, the 90-day complication rates are similar across all organ groups.

## Introduction

The Annual Data Report of the US Organ Procurement and Transplantation Network and the Scientific Registry of Transplant Recipients for 2024 revealed a record breaking increase in the number of solid organ transplantations. A striking increase in the number of heart (73%), lung (54%), liver (52%) and kidney (52%) transplants is noted between 2012 and 2022 [1]. Similarly, Germany has seen an upward trend, with an 8.1% increase in the number of transplanted organs in 2023 compared to 2022 [2]. Moreover, alongside the increase in transplant numbers, there has been a significant improvement in the life expectancies of solid-organ transplant (SOT) patients in recent years. The 5-year patient survival rates have improved, currently ranging from 60.5% for lung transplant recipients to 81% for heart transplant recipients, 81.7% for liver transplant recipients and 88.2% for kidney transplant recipients [3].

With the rising figures in both the number of transplantations performed and post-transplant survivorship, there is an increasing need for total hip arthroplasty (THA) and total knee arthroplasty (TKA) in SOT patients [4].

The discussion surrounding the risks associated with total joint replacement (TJR) in SOT patients encompasses various factors, such as immunosuppression, infection and postoperative complications. In recent years, several new studies have demonstrated acceptable perioperative risk in this patient population [5–7] which is in contrast with the reported outcomes from several years ago, when elective surgeries were associated with significantly higher risks for complications such as infections [8–10]. In patients with SOT, immunosuppression increases susceptibility to infections and impairs wound healing. Mammalian target of rapamycin (mTOR) inhibitors, while essential for preventing organ rejection, have been associated with impaired post-surgical wound healing [11]. Similarly, anti-thymocyte globulin, a polyclonal antibody, and mycophenolate, an antimetabolite that inhibits purine synthesis, suppress the immune system, thereby reducing the body’s ability to fight infections and impairing wound healing [12]. Additionally, chronic steroid use can diminish bone quality, thereby increasing the risk of periprosthetic fractures. The long-term use of immunosuppressive drugs to prevent organ rejection further exacerbates these risks [13, 14]. Additionally, transplant recipients often have multiple comorbidities, such as diabetes, cardiovascular disease and renal dysfunction, which can complicate perioperative management and further increase infection risks [15].

The aim of this study is to report on 1) implant survivorship, 2) patient survivorship, and 3) complication rates for knee and hip arthroplasty performed in patients with solid organ transplants, including subgroups with heart, lung, liver and kidney transplants. Our hypothesis is that TJR has good mid-term survivorship and is safe in this highly vulnerable population.

## Materials and methods

### Study design

Patients with SOT defined as heart, lung, liver or kidney transplant, who underwent primary THA or TKA at an academic institution from January 1, 2013, to July 31, 2023, were retrospectively identified in our prospective organized digital medical chart system. Further inclusion criteria were age > 18 years old and a minimum of one year of follow-up. Patients with incomplete medical records, oncologic indications, femoral neck fractures, revision joint arthroplasty, and previous osteosynthesis of the hip/knee were excluded. Data were obtained from the patients’ electronic medical records. Ethical approval was obtained from the local ethics committee (Ethikkommission der Medizinischen Fakultät der Ludwig-Maximilians-Universität, reference number 23–0826).

### Data collection

Data was retrospectively collected from a prospectively maintained database, and included demographic characteristics, pertinent transplant data including solid organ type and immunosuppression regimen, intraoperative and postoperative data. Both early (defined as occurring within 4 weeks following surgery) and late complications were collected. Postoperative complications were categorized using the Clavien-Dindo classification [16]. Perioperative blood loss was estimated by measuring the contents in the suction canister and weighing the blood-soaked gauzes. A restrictive transfusion strategy (hemoglobin < 7 g/dL or symptomatic anemia with hemoglobin > 7 g/dL) was applied for allogenic red blood cell transfusions.

Clinical and radiological follow-up were conducted annually, and all patients were contacted for the final follow-up. Radiographs were evaluated for radiolucent lines and signs of loosening. Implant survivorship, with revision surgery as the endpoint, and reoperation for any reason were documented. Implant survivorship and reoperation-free survival were defined as the time from prosthesis implantation to revision and reoperation for any cause, respectively. For deceased patients, the implant status at the time of death was obtained from medical records or confirmed by the family.

### Patient demographics and study groups

**Fig. 1:**
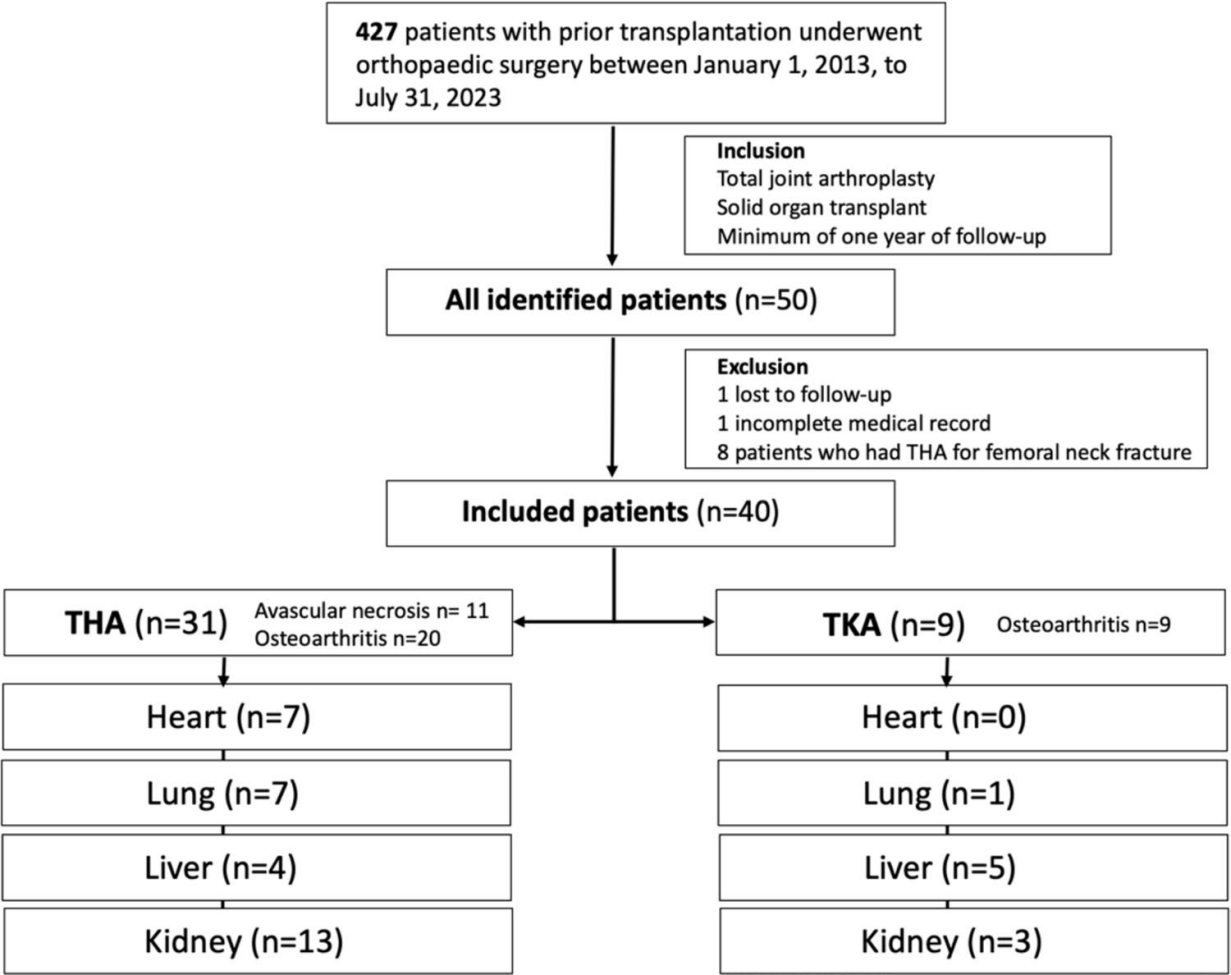
Flow chart demonstrating the inclusion and exclusion criteria and the subgroups

Of the 427 patients with SOT that underwent musculoskeletal surgery in the pre-defined ten year period, fifty underwent either THA or TKA and were eligible for this study based on the inclusion criteria. Of these patients, one was lost to follow-up, one was excluded due to incomplete medical record data, and eight were excluded because their THA was performed for a femoral neck fracture, leaving 40 patients for analysis (Fig. 1).

The distribution of transplant types was 7 heart transplants, 8 lung transplants, 9 liver transplants and 16 kidney transplants. The mean time from transplant to arthroplasty surgery was 9.74 years (0.3–30 SD = 6.68) years and the mean follow-up time was 5.18 years (1.2–10, SD = 2.45).

THA was performed in 31 patients and TKA was performed in 9 patients.

The indications for THA were avascular necrosis in 11 cases (35.5%, 11/31) and osteoarthritis in 20 cases (64.5%, 20/31), while all TKAs were performed for end-stage knee osteoarthritis.

Patients' demographics are summarized in Table 1.

**Table 1:** Patient demographics

	All (n = 40, 100%)	Heart (n = 7, 17.5%)	Lung (n = 8, 20%)	Liver (n = 9, 22.5%)	Kidney (n = 16, 40%)	p-value
Mean Age (y)	60.9 (38–82, SD = 10.2)	60.7 (45–73, SD = 10.4)	63.4 (51–73, SD = 6.9)	61.7 (45–82, SD = 12.2)	59.3 (38–75, SD = 11)	n.s
Mean BMI (Kg/m^2^)	26.4 (16.8–36.7, SD = 4.6)	27.4 (22.1–33, SD = 4)	23.4 (16.8–29.8, SD = 4.1) *	26.2 (18.1–33.4, SD = 5.4)	27.5 (20.6–36.7, SD = 4.6)	*0.0387
Gender Female Male	18 (45%) 22 (55%)	0 (0%) 7 (100%)	5 (62.5%) 3 (37.5%)	3 (33.3%) 6 (66.6%)	10 (62.5%) 6 (37.5%)	-
Anticoagulants	21/40 (52.5%)	4/7 (57.1%)	5/8 (62.5%)	2/9 (22.2%)	10/16 (62.5%)	-
ASA score	3.1 (2–4)	3.00	3.1 (3–4)	3.1 (3–4)	3.1 (2–4)	n.s
CCI	3.5 (1–6, SD = 1.2)	3.1 (2–4, SD = 0.7)	3.8 (2–5, SD = 1)	3.6 (1–5, SD = 1.3)	3.5 (1–6, SD = 1.3)	n.s
Median follow up (y)	5.2 (1.2–10, SD = 2.5)	7 (1.2–10, SD = 2.6)	4.6 (2–8.9, SD = 2.3)	4.1 (1.6–10, SD = 2.7)	5.1 (2–10, SD = 2.3)	n.s

Bold values indicate statistically significant differences compared to other groups

*ASA* American society of Anesthesiologists, *BMI* body mass index, *CCI* Charlson comorbidity index, *y* years

*Represents statistical significance, p < 0.05

### Surgical technique

All THA surgeries were performed by 6 arthroplasty-trained surgeons using a direct lateral approach in 11 cases and a direct anterior approach (DAA) in 20 cases. The implants were cemented in 15 cases and uncemented in 16 cases.

TKA were performed by 5 arthroplasty-trained surgeons without a tourniquet, using a standard medial parapatellar approach.

For both TKA and THA, antibioprophylaxis consisted of a third-generation cephalosporins administered over 24 h. All patients received 1 g of intravenous tranexamic acid (TXA) 30 min before the incision in the absence of contra-indications. Physical therapy began on the day of surgery.

### Data analyses

Normally distributed, continuous variables are presented as means ± standard deviation and were compared using a student’s t-test of ANOVA. Non-normally distributed continuous data are reported as median (range) and compared with Mann Whitney U test or Kruskal Wallis test.

In the analysis of complications across different organ groups, Fisher's Exact Test was used to assess the distribution of all Clavien-Dindo complication grades (0 to 5), as well as to conduct a separate comparison between major (grade ≥ 3) and minor (grade ≤ 2) complications across the organ groups.

A P value of < 0.05 was considered statistically significant (* p < 0.05, ** p < 0.01). Kaplan–Meier survivorship curves were used to demonstrate overall patient survivorship for the entire cohort and the subgroups, as well as re-operation free implant survival for the entire cohort and the organ specific subgroups. Comparisons between groups were tested using a log-rank test.

All statistical analyses were run in GraphPad Prism 10.1.2 (GraphPad Software LLC, Boston, Massachusetts USA) and R Statistical Software (Version 2024.04.2 for macOS, R Core Team 2022, Vienna, Austria).

## Results

### Perioperative data

Perioperative data are summarized in Table 2.

**Table 2:** Surgical patient data

	All (n = 40)	Heart (n = 7)	Lung (n = 8)	Liver (n = 9)	Kidney (n = 16)	p-value
THA/TKA	31/9 (77.5%/22.5%)	7/0 (100%/0%)	8/0 (100%/0%)	5/4 (55.5%/44.5%)	14/2 (87.5%/12.5%)	–
Operation time (min)	96.61 (34–190)	91 (66–118)	108.1 (66–186)	107.2 (51–190)	85.6 (34–142)	n.s
Estimated blood loss (ml)	545.9 (50–1600)	580 (300–1100)	437.5 (200–800)	566.7 (100–1600)	580 (50–1400)	n.s
Hemoglobin (mg/dl) Preoperative Day 1 Discharge	12.9 (9.4–18.4) 9.9 (7.5–13.5) 9.8 (7–12.5)	14 (10.2–18.4) 10.6 (8.2–13.5) 10.3 (8.3–12.4)	12.1 (10.4–13.3) ** 9.2 (7.8–10.1) * 9.1 (7–10.2) **	14.1 (11.7–16.9) 10.8 (8–12.5) 10.9 (9.7–12.5)	12.1 (9.4–15.3) * 9.5 (7.5–12.4) * 9.3 (7.8–12.4) **	**0.01/*0.0126 *0.0158/* 0.0414 ** 0.0017/** 0.0052

Bold values indicate statistically significant differences compared to other groups

The mean length of stay was 9.8 days (6–21, SD = 4.43). Of the patients, 28/40 (70%) were discharged to a rehabilitation clinic, and 12/40 (30%) were discharged home for further ambulatory rehabilitation

*Min* minutes, *ml* milliliter, *dl* deciliter

*Represents statistical significance, p < 0.05

**Represents statistical significance, p < 0.01

### Implant survivorship

**Fig. 2:**
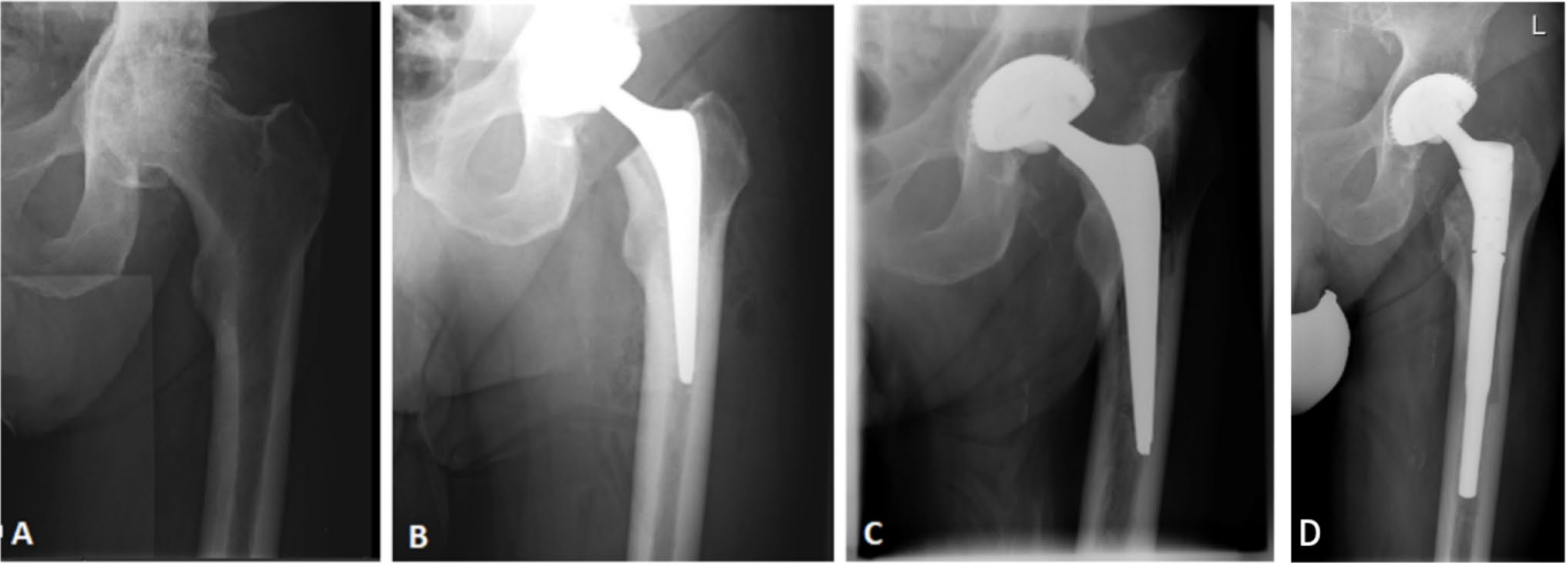
**A** Pre-operative **B** 6 weeks post-operative **C** 9 years post-operative and **D** post-revision anteroposterior radiographs of the left hip of a 74-year-old male with a renal transplant. The revision surgery was performed nine years after the initial procedure due to aseptic loosening

The implant survivorship and reoperation-free survival of the entire cohort at last follow up were 97.5% and 85%, respectively. At 5.18-years mean follow-up (1.2–10, SD = 2.45) there were six reoperations (15%, 6/40), including one revision for aseptic loosening of a THA 9 years after the initial surgery (Fig. 2).

In the TKA group, two patients (22%, 2/9) required reoperation. One patient on anticoagulant therapy underwent surgical evacuation of a hematoma and debridement, antibiotics, and implant retention (DAIR) procedure one week after the initial surgery, and another patient required reoperation for deep fascial dehiscence 11 months postoperatively. Perioperative cultures taken during these surgeries were negative.

In the THA group, there were four reoperations, including one revision. Two patients (6.5%, 2/31) underwent a DAIR procedure for acute periprosthetic joint infection (PJI) with *Staphylococcus haemolyticus* 16 days post index surgery and *Enterococcus faecium* 20 days postoperative. One patient (3.3%, 1/31) required reoperation for delayed wound healing. Both patients with PJI remained infection-free following the DAIR procedure and a 12-week course of antibiotic therapy. One patient passed away three years postoperatively due to lung transplant failure, while the other remains highly satisfied and underwent a complication-free contralateral THA six years after the initial surgery.

**Fig. 3:**
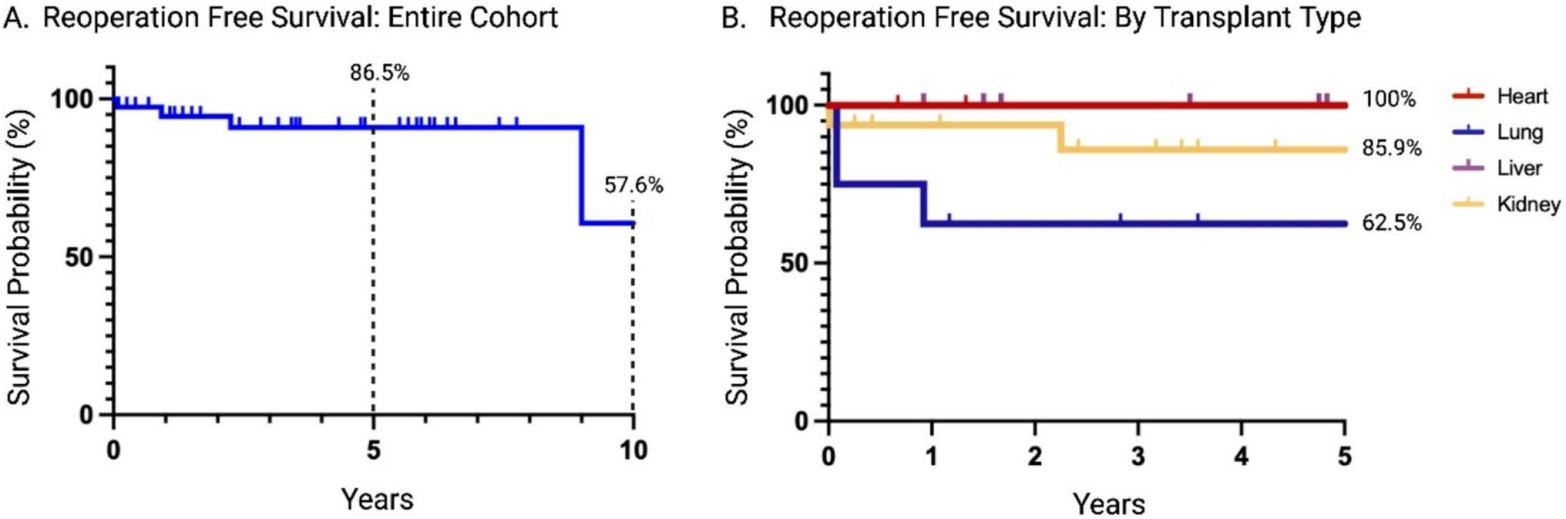
**A** Kaplan–Meier survivorship curve representing the overall implant reoperation-free (%) survival rate after total joint arthroplasty. **B** Kaplan–Meier survivorship curve representing subclasses of solid organ transplant patient`s implants reoperation free survivorship in percent

Kaplan Meier survival estimates demonstrated a 5- and 10-year reoperation free survival of the entire cohort of 86.5% (95% CI: 76%–98.4%) and 57.6% (95% CI: 25.6%–100%), respectively. The 5-year reoperation free survival varied across transplant groups. None of the patients in the heart transplant or liver transplant group experienced a reoperation at 5 years. The kidney transplant and lung transplant patients had a 5-year reoperation free survival of 85.9% (95% CI: 69.5%–100%) and 62.4% (95% CI: 36.5%–100%), respectively. Although the lung transplant group had the shortest reoperation-free survival, this was not statistically significant (p = 0.07), however; there was a significantly higher risk of reoperation in these patients with a HR of 6.9 (95% CI: 1.1–41.2, p = 0.035), indicating a higher likelihood of needing reoperations compared to other groups. (Fig. 3).

### Patient survivorship

At 5 and 10 years follow-up, the overall survival rates were 92.5% and 85%, respectively. Of the initial cohort of 40 patients, six patients had died due to medical and transplant-related complications unrelated to TJA surgery.

**Fig. 4:**
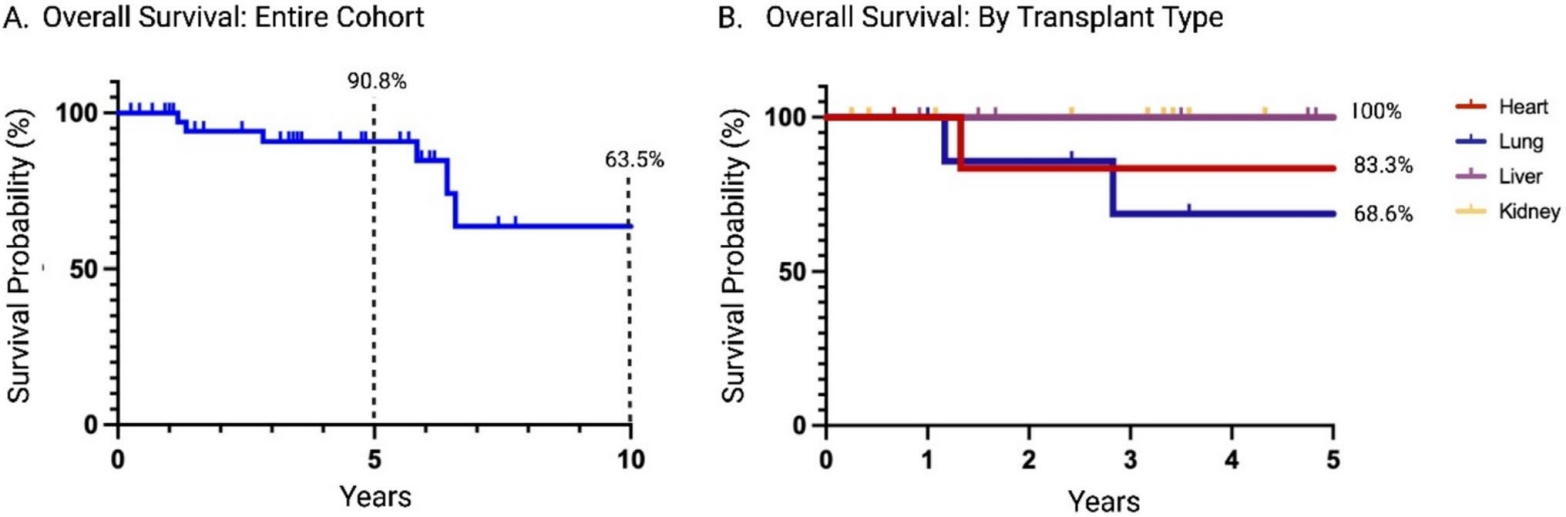
**A** Kaplan–Meier survivorship curve representing overall solid organ transplant patient survivorship after total joint arthroplasty as percent survival **B** Kaplan–Meier survivorship curve representing percent survival of subclasses of solid organ transplant patients after total joint arthroplasty

Kaplan–Meier survival estimate demonstrated a 5- and 10-year survival of 90.8% (95% CI 81.3%–100%) and 63.5% (95% CI 41.1%–98.3%), respectively (Fig. 4A). The 5-year survivorship varied among transplant cohorts, with the liver and kidney cohort achieving 100% survivorship, the heart transplant group demonstrating 83.3% survivorship, and the lung transplant recipients exhibiting the lowest 5-year patient survivorship at 68.6% (p = 0.04) (Fig. 4B). Additionally, the risk of death after TJA was significantly higher in lung transplant recipients with a HR of 7.04 (95% CI: 1.2–45.5, p = 0.03). (Fig. 4).

### Complications

There were 20 early complications in this cohort. The detailed list of the complications, classified according to the Clavien-Dindo classification to evaluate perioperative intrahospital complications, is illustrated in Table 3.

**Table 3:** This table shows the number of complications: twelve type 2 complications were found in nine patients

Clavien-Dindo Classification	All (n = 40)	Heart (n = 7)	Lung (n = 8)	Liver (n = 9)	Kidney (n = 16)
0	20	4	2	7	7
1 Hypokalemia	3 3				
			1		2
2 Malnutrition Symptomatic Anemia UTI Gastroenteritis	12 3 8 1 0	2	1 1 1	2	5
3a Thrombosis	1 1				
					1
3b Acute PJI Hematoma	3 2 1		2		
					1
4 Septic schock	1 1				
		1			
5	0				

*PJI* Periprosthetic joint infection, *UTI *Urinary tract infection

**Fig. 5:**
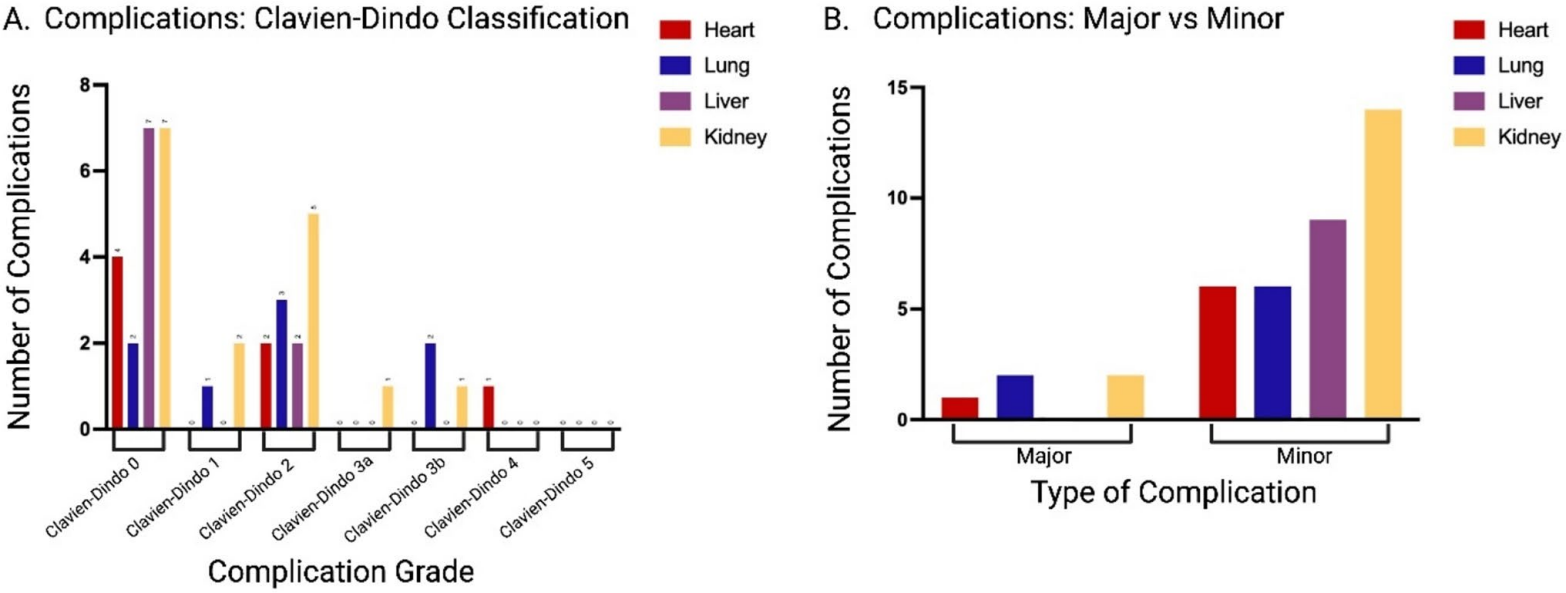
**A** Number of complications per subgroup according to Clavien-Dindo-Classification 0–5. **B** Number of major (grade ≥ 3) and minor (grade ≤ 2) complications per subgroup according to Clavien-Dindo-Classification

There was no difference between the distribution of complications (p = 0.5), nor was there a difference between the number of major versus minor complications (p = 0.2) across the different solid organ transplant groups. (Fig. 5).

Regarding the management of complications, four patients required early surgery for PJI with DAIR, and one patient required superficial wound revision due to delayed wound healing and hematoma.

Furthermore, there were two late complications: one required reoperation, and the other necessitated revision surgery.

## Discussion

The key finding of this study is the exceptional mid-term implant survivorship of 97.5% following TJA in patients after SOT. This high rate of implant survivorship is encouraging, as it demonstrates that SOT recipients can achieve favorable outcomes despite their complex medical conditions. Notably, lung transplant recipients have the lowest rates of both reoperation-free survival and patient survivorship compared to heart, kidney and liver transplant recipients. However, the 90-day complication rates are similar across all organ groups.

### Implant survivorship

This study found an excellent implant survivorship of 100%, at both one and five years. Furthermore, at last follow-up, there were 6 (15%, 6/40) reoperations including only one revision surgery (2.5%, 1/40).

Our results are similar to the published literature on the same subject, with implant survivorship ranging from 95% to 95.6% at 1–2 years and 92.1% to 94% at 5 years, with no significant difference between organ groups [6, 17].

Gupta et al. found that SOT patients experienced higher rates of 90-day all-cause revisions and periprosthetic joint infections within 10 years compared to the general population undergoing TKA. However, there was no significant increase in all-cause revision rates at 2, 5, and 10 years [18].

Despite these encouraging findings, the implant survivorship in the SOT population remains lower compared to TJA in non-transplanted patients, where TKA implant survivorship is 99% at 10 years and THA is 99.6% at 5 years and 98.7% at 10 years [19, 20].

### Patient survivorship

At last follow-up overall patient survivorship was 85%, with the lowest survivorship of 62.5% found in the lung transplantation group.

Our results are similar to the study by Wu et al., which reported an overall mortality of 2.9% and 23.2% at one and four years, respectively, with the highest mortality observed in the lung transplant group [6]. Lung transplant recipients exhibited worse overall survival and reoperation-free survival compared to heart, liver and kidney transplant recipients. Several factors may contribute to this finding. Lung transplant recipients have worse survival at baseline compared to other solid organ transplant recipients [21]. Additionally, lung transplant recipients frequently suffer from chronic lung allograft dysfunction, such as bronchiolitis obliterans syndrome, which impairs their respiratory function and overall physiological reserve, making them more prone to postoperative complications and reoperation [22]. Poor physiological reserve makes patients more vulnerable to postoperative complications, including symptomatic anemia, cardiopulmonary events, hypoxia, respiratory failure, and infections. These complications can impair wound healing, increase the risk of PJI, and elevate the likelihood of falls, all of which may necessitate re-operation. Lung transplant patients also tend to have more comorbidities and a higher perioperative risk, further explaining the higher mortality and reoperation rates observed in this group [21, 23]. Lastly, lung transplant recipients are at higher risk of graft failure and chronic rejection, contributing to long-term complications and poorer surgical outcomes [8, 23, 24].

However, retrospective studies by Leford et al. and Chalmers et al., revealed the highest mortality in renal transplant patients after TJA with no significant difference between organ groups and overall mortality rates of 1.9% at one year and 13.3% at five years [8, 17].

The mortality rates reported in this study are significantly higher than those observed in our practice for primary TJA in a similar age group, where the mortality rates are 1% at five years and 4% at ten years, respectively [20].

### Complications

In this cohort, the complication rate was 50%, with 31.5% classified as major and 69.5% as minor. This is consistent with other studies such as Ledford et al.’s retrospective also reported high complication rates (29–33%) and reoperation rates (7.2–9.1%) [8]. A major concern in this population is PJI, with earlier studies reporting infection rates ranging from 6.8 to 17.3% after TJA in SOT patients [25, 26]. However more recent data such suggest that the risk of infection may have decreased. For instance, Wu et al. demonstrated a 2.7% rate of PJI, while we found a 5% in this cohort. Furthermore, Gupta et al. found no increased risk of PJI when compared to both matched and unmatched controls. Another controlled study reported no significant difference in readmission or infection rates in the SOT group [6, 18, 26]. These decreased infection rates may be attributed to improved perioperative management, including better infection prevention strategies, more effective use of perioperative antibiotics and advancements in surgical technique. Nevertheless, SOT recipients still face a higher risk of infection compared to non-transplant patients undergoing TJA.

Our study did not reveal statistically significant differences in complication rates across different transplant orgran groups which is consistent with the findings by Klement et al. [14]. However, these results contradict the findings of Klatt et al. and Duplantier et al., who reported increased infection rates and complications in the renal transplantation group compared to the non-renal transplantation group and liver transplantation group, respectively [13, 23].

Variations in reported complication rates and the vulnerability of specific transplanted organs to complications could be explained by their inconsistent report across studies, with discrepancies in the timing (short-term versus short- and long-term) and the classification of complications (medical versus implant-related, minor versus major).

When considering all 90-day complications, reported rates for primary TJA in the non-transplanted population range from 3.8% to 5.5% which is significantly lower than in the SOT population [24, 27]. This was confirmed by Wu et al. who found 43% complications in the transplanted patients versus 17% in the non transplanted group at two years of follow-up [6], similar to the 50% found in this study.

The higher complication and infection rate, as well as the lower implant survivorship seen in this population can be explained by multiple factors such as lifetime use of immunosuppression medication, impairing wound healing and increasing the risk of infection, and anemia a recognized risk factor for PJI [28]. Morevoer, chronic steroid use induces poor bone quality thus increases the risk of implant related complications [5]. Finally, one of the main risks for increased complications is associated with the greater burden of comorbidities commonly found in SOT patients [18].

However, despite the elevated risk of complications, studies demonstrates high patient satisfaction and good functional outcomes after TJA in SOT patients [7]. This study is not without limitations. First, the cohort size is relatively small, which limits the statistical power to detect potential differences in outcomes across different SOT types. However, while few studies report on outcomes between different SOT types, most existing studies on TJA in SOT patients also include limited patient numbers. Furthermore the possibility of a type 2 error cannot be excluded as the small number of complications observed in this cohort makes it unlikely to detect differences that may actually exist between organ groups.

## Conclusion

With a mean follow-up of 5.2 years, patients with SOT exhibit excellent implant survivorship, with a rate of 97.5%. Moreover, lung transplant recipients show the lowest rates of both reoperation-free survival and overall patient survivorship compared to heart, kidney and liver transplant recipients. Despite this, the 90-day complication rates are similar across all organ groups. To mitigate complications, it is crucial that SOT recipients receive care from specialized multidisciplinary teams.

## Data Availability

The data that support the findings of this study are available from the corresponding author upon reasonable request.
